# Molecular cloning and functional analysis of 4-Coumarate:CoA ligase 4(*4CL-like 1*)from *Fraxinus mandshurica* and its role in abiotic stress tolerance and cell wall synthesis

**DOI:** 10.1186/s12870-019-1812-0

**Published:** 2019-06-03

**Authors:** Xiaohui Chen, Hengtao Wang, Xiaoyi Li, Kai Ma, Yaguang Zhan, Fansuo Zeng

**Affiliations:** 1 State Key Laboratory of Tree Genetics and Breeding (Northeast Forestry University), Harbin, 150040 China; 2 College of Life Science, Northeast Forestry University, Harbin, 150040 China

**Keywords:** *Fraxinus mandshurica*, *4CL/4CL-like* gene, Lignin, Cell wall, Osmotic stress

## Abstract

**Background:**

Four-Coumarate:CoA ligase gene (*4CL*) plays multiple important roles in plant growth and development by catalyzing the formation of CoA ester. *4CL* belongs to the plant phenylpropane derivative, which is related to the synthesis of flavonoids and lignin and is a key enzyme in the biosynthetic pathway.

**Results:**

In this study, 12 *4CL* genes of *Fraxinus mandschurica* were identified and named *Fm4CL1*-*Fm4CL12*, respectively. The analysis of the expression pattern of *Fm4CL* genes indicate that *Fm4CL-like 1* gene may play a role in the lignin synthesis pathway. Our study indicate that overexpression of *Fm4CL-like 1* increases the lignin content of transgenic tobacco by 39.5% compared to WT, and the S/G ratio of transgenic tobacco increased by 19.7% compared with WT. The xylem cell layer of transgenic line is increased by 40% compared to WT, the xylem cell wall thickness increased by 21.6% compared to the WT. Under mannitol-simulated drought stress, the root length of transgenic tobacco is 64% longer than WT, and the seed germination rate of the transgenic lines is 47% higher than that of WT. In addition, the H_2_O_2_ content in the transgenic tobacco was 22% lower than that of WT, while the POD and SOD content was higher than WT by 30 and 24% respectively, which showed *Fm4CL-like 1* affect the accumulation of reactive oxygen species (ROS). The MDA content and relative conductivity was 25 and 15% lower than WT, respectively. The water loss rate is 16.7% lower than that of WT. The relative expression levels of stress-related genes *NtHAK*, *NtAPX*, *NtCAT*, *NtABF2*, and *NtZFP* were higher than those of WT under stress treatment. The stomatal apertures of OE (Overexpression) were 30% smaller than those of WT, and the photosynthetic rate of OE was 48% higher than that of WT. These results showed that the overexpression line exhibited stronger adaptability to osmotic stress than WT.

**Conclusions:**

Our results indicate that *Fm4CL-like 1* is involved in secondary cell wall development and lignin synthesis. *Fm4CL-like 1* play an important role in osmotic stress by affecting cell wall and stomatal development.

**Electronic supplementary material:**

The online version of this article (10.1186/s12870-019-1812-0) contains supplementary material, which is available to authorized users.

## Background

Lignin is second largest polymer in plants after cellulose [1, 2]. The xylem of the plant contains a large amount of lignin (about 25% of the woody part), mainly in the middle of the cellulose fiber. Lignin has several important functions for plants. It is the most important carbon storage method in the biosphere and is the main polymer in the cell wall [3], increasing cell wall hardness and enhancing the mechanical support and compressive strength of cells [4–6]. The formation of this tissue provides plant mechanical support [7, 8]. Lignin enhances plant defense against biotic and abiotic stresses [9, 10].

Lignin is a complex phenolic polymer mainly composed of lignitols such as coumarin, coniferyl alcohol, and sinapicol [11, 12], a complex polymer of phenylpropane monomers [13]. Lignin can be divided into three types, according to its constituent monomers: lilacyl-lignin (S-lignin) comprised of lilac-based propane structural monomers; guaiacyl-lignin (G-lignin) comprised of from guaiacylpropane structural monomers, and p-hydroxyphenyl lignin (H-lignin) comprised of p-hydroxy phenylpropane structural monomer [11]. Among these, H-lignin is mainly found in grasses, S-lignin is mainly found in angiosperms, and G-lignin is widely found in gymnosperms and angiosperms [5, 11]. The three kinds of lignin in nature do not occur in isolation in the plant, the gymnosperms mainly contain guaiacyl-based lignin (G); dicotyledons mainly have guaiacyl-lilac-based lignin (G-S); while monocots have conjugated Guajiba-lilacyl-p-hydroxyphenyl lignin (G-S-H) [11, 14, 15]. In addition, the activity and ratio of the different enzymes involved in lignin biosynthesis determine the type and rate of monomer synthesis and the proportion of each lignin monomer [16, 17].

The phenylpropane pathway is an important step in the lignin biosynthesis. 4-coumarate: CoA ligase (4CL) is a key rate-limiting enzyme in the phenylpropanoid metabolism of plants [18], which belongs to the plant phenylpropane derivatives involved in the synthesis of flavonoids and lignin [19]. 4CL uses cinnamic acid and its hydroxyl or methoxy derivatives p-coumaric acid, caffeic acid, ferulic acid, 5-hydroxyfreulic acid, and sinapic acid as substrates to produce their coenzyme A esters [12, 20]. It is located at the crossover point in the metabolic pathway of phenylpropanoids to branching metabolic pathways such as those of flavonoids, lignin, and cinnamate [21], catalyzing the production of different types of plant resistance-related substances [22, 23]. The *4CL* gene family is present in most vascular plants [24].

4CL genes can be divided into two broad categories. Class I *4CL* genes, including those of most dicotyledonous plants, such as *Arabidopsis At4CL1* and *At4CL2*, *Populus Pt4CL1*, and *soybean Gm4CL2*, are associated with the biosynthesis of lignin and other phenylpropanoid derivatives [25–27]. Class II includes the *4CL* genes of monocotyledonous plants and gymnosperms, as well as the *4CL* genes of some dicotyledonous plants, such as *Arabidopsis At4CL3*, *Pt4CL2*, and *rice 4CL*, which are involved in the production of plant flavonoids and antitoxins [28–31]. Some additional genes contain the same conserved motifs as *4CLs* and have high homology with the proteins encoded by *4CLs* [26]. These are classified as *4CL-like* and their functions are not yet clear, but may be similar to the *4CL* function in leaves [24]. Both *4CL* and *4CL-like* genes control the plant’s various physiological functions and improve the ability of the plant to adapt to the environment.

*Fraxinus mandshurica Rupr*. belongs to the genus *Fraxinus*, one of the three hardwood broadleaf tree families in northeastern China [32], and is a dioecious, wind-dispersed, cold-adapted susceptible to drought and saline-alkali stress. *Fraxinus mandshurica* has become China’s most endangered tree species [33] and was given national secondary protection status. At the same time, *Fraxinus mandshurica* has very good overall strength properties, its anti-vibration strength and steam bending strength are excellent, leading to its high economic value in forestry production.

Drought stress lead to irreversible changes in the plant cell membrane and seriously affect the plant development and crop yield. Cell membrane permeability is changed, leading to the loss of a large number of dissolved substances and a serious water shortage will lead to plant cell protein denaturation, affecting photosynthesis [34]. Drought stress can also produce a large number of plant reactive oxygen species, such as hydroxyl radicals, superoxide anion, and hydrogen peroxide [35], Under stress, the dynamic balance of active oxygen production and removal in plants is destroyed, which leads to an increase in membrane lipid peroxidation products and the destruction of cell membrane integrity and permeability [36]. Research has shown that abiotic stress leads to the rapid activation of *4CL* expression [37], indicating that *4CL* plays a role in the anti-retrogradation pathway. It has been hypothesized that the function of *4CL* is closely linked to the environmental stresses experienced by plants [38]. Therefore, increasing the expression level of *4CL* may be an effective method to increase plant stress resistance. In this study, 12 *Fm4CL/4CL-like* genes were identified from *Fraxinus mandshurica*, and their temporal and spatial expression patterns were characterized. *Fm4CL-like 1* was also cloned and its overexpression in tobacco increased the lignin content and the resistance of the transgenic tobacco to mannitol-induced drought stress compared to the wild type (WT).

## Results

### Identification and phylogenetic analysis of *4CL* from *Fraxinus mandschurica*

**Fig. 1:**
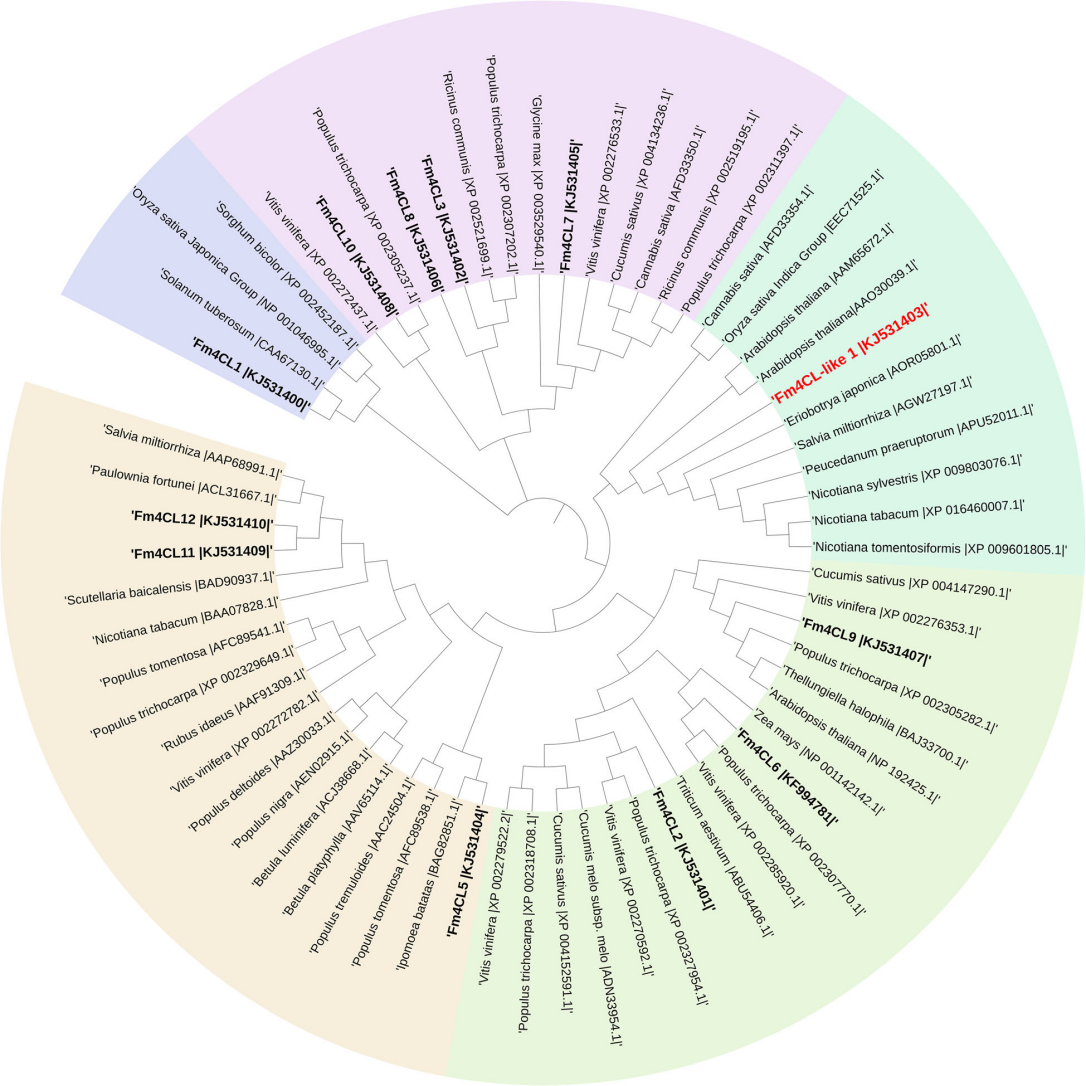
Phylogenetic analysis of *Fm4CL* genes. The alignment was constructed using the ClustalW method and the phylogenetic tree was constructed with the Fm4CL proteins sequences obtained from the NCBI database (http://www.ncbi.nlm.nih.gov) using the neighbor-joining (NJ) method with MEGA7 software

We identified 12 *4CL* genes from the *Fraxinus mandshurica* transcriptome databases using the BioEdit local Blast method with 5 *4CL* genes of *Arabidopsis thaliana* and 3 *4CL* genes of *Populus trichocarpa* nucleotide sequences retrieved from NCBI, named *Fm4CL1- Fm4CL12*, which have all been uploaded to GenBank and assigned accession numbers (KJ531400- KJ531404, KF994781, KJ531405-KJ531410), characteristics of 12 *Fm4CL* gene sequences are in Additional file 1: Table S1. To understand the evolutionary relationship of 4CL proteins between *Fraxinus mandshurica* and other species, we collected the sequences of 53 4CL proteins from *Populus*, *Oryza sativa*, *Arabidopsis*, and other species and constructed phylogenetic trees (Fig. 1). The 53 4CL proteins could be divided into five broad categories. *Fm4CL3, Fm4CL7, Fm4CL8,* and *Fm4CL10* had higher homologies with *4CL* sequences from *Vitis vinifera, Populus,* and *Arabidopsis*. *Fm4CL2*, *Fm4CL6*, and *Fm4CL9* had high homology with sequences from *Cucumis sativus, Zea mays, Vitis vinifera,* and *Populus*. *Fm4CL5*, *Fm4CL11*, and *Fm4CL12* had high homology with sequences from *Betula luminifera, vitis vinfera*, and *Salvia miltiorrhiza*. The phylogenetic analysis demonstrate that *Fm4CL4* and its orthologs in *Arabidopsis*, *Nicotiana sylvestris*, *Nicotiana tabacum*, *Oryza sativa*, *Salvia miltiorrhiza* belong to the *4CL-like* gene subfamily, so we named it *Fm4CL-like 1*. This indicates that *Fm4CLs* is closely related to these species.

### Expression analysis of the *Fm4CL/4CL-like* gene family

**Fig. 2:**
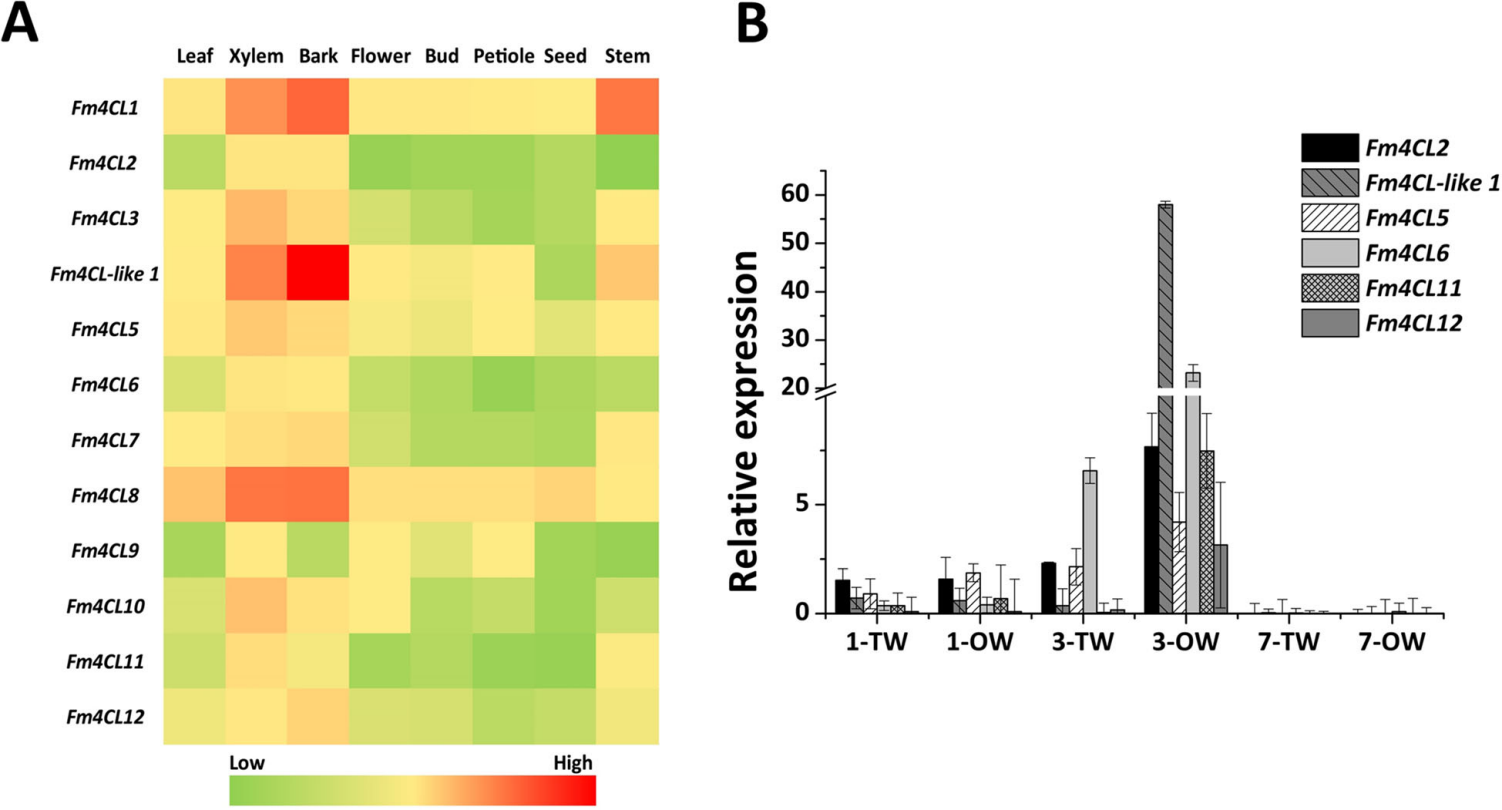
(**a**) Transcript levels of *Fm4CL* genes in different tissues of *Fraxinus mandshurica*. Tissue expression analysis of 12 *Fm4CL genes* in the leaf, xylem, bark, flower, bud, petiole, seed, and stem of the *Fraxinus mandshurica* plant. Red indicates high gene expression, green indicates low gene expression, and yellow indicates moderate gene expression. (**b**) qRT-PCR values of *Fm4CLs/4CL-like* genes in *Fraxinus mandshurica* tension wood. *Fm4CL2*, *Fm4CL-like-1*, *Fm4CL5*, *Fm4CL6*, *Fm4CL11*, and *Fm4CL12* are expressed in the *Fraxinus mandshurica* plant. CK, TW, and OW indicate untreated as a control, tension wood, and corresponding wood, respectively.

To assess the differential expression pattern of the *Fm4CL* genes family in different tissues of *Fraxinus mandshurica*, samples of leaves, xylem, bark, flowers, bud, stem, petiole and seeds were collected in May. The qRT-PCR results showed that 12 *Fm4CL* transcripts could be detected in different tissues of *Fraxinus mandshurica* and has obvious tissue specific expression (Fig. 2a). *Fm4CL2*, *Fm4CL6*, *Fm4CL9* and *Fm4CL11* were expressed at low levels in all the tissues and organs of *Fraxinus mandshurica*. Among 12 *4CL/4CL-like* genes, *Fm4CL-like 1* expression was the highest in bark, while *Fm4CL-like 1* and *Fm4CL8* were expressed at the highest level in xylem, indicating that *Fm4CL8* and *Fm4CL-like 1* may be involved in the biosynthesis of xylem.

*Fraxinus mandshurica* was subjected to tension treatment. Control check (CK), Tension Wood (TW), and Opposite Wood (OW) indicate untreated samples, tension stress surface samples, and corresponding surface samples, respectively (Fig. 2b). The expression of *Fm4CL/4CL-like* genes in 24 h-treated *Fraxinus mandshurica* tension wood (TW) was almost unchanged, but the expression in OW samples was generally higher than that in TW. After 3 d treatment, the expression level of *Fm4CL* genes in OW was significantly higher than TW. The *Fm4CL* genes were almost undetectable in *Fraxinus mandshurica* tension treated wood at 7 d. The expression pattern of the *Fm4CL* genes in tension wood differed, but all genes responded to the tension treatment. Among them, *Fm4CL-like 1* had the highest expression level in OW after 3 d treatment, it was 3~20 times more than other genes, which indicated that *Fm4CL-like 1* might be involved in the synthesis of cell wall and lignin.

### Overexpression of *Fm4CL-like 1* gene in transgenic tobacco

**Fig. 3:**
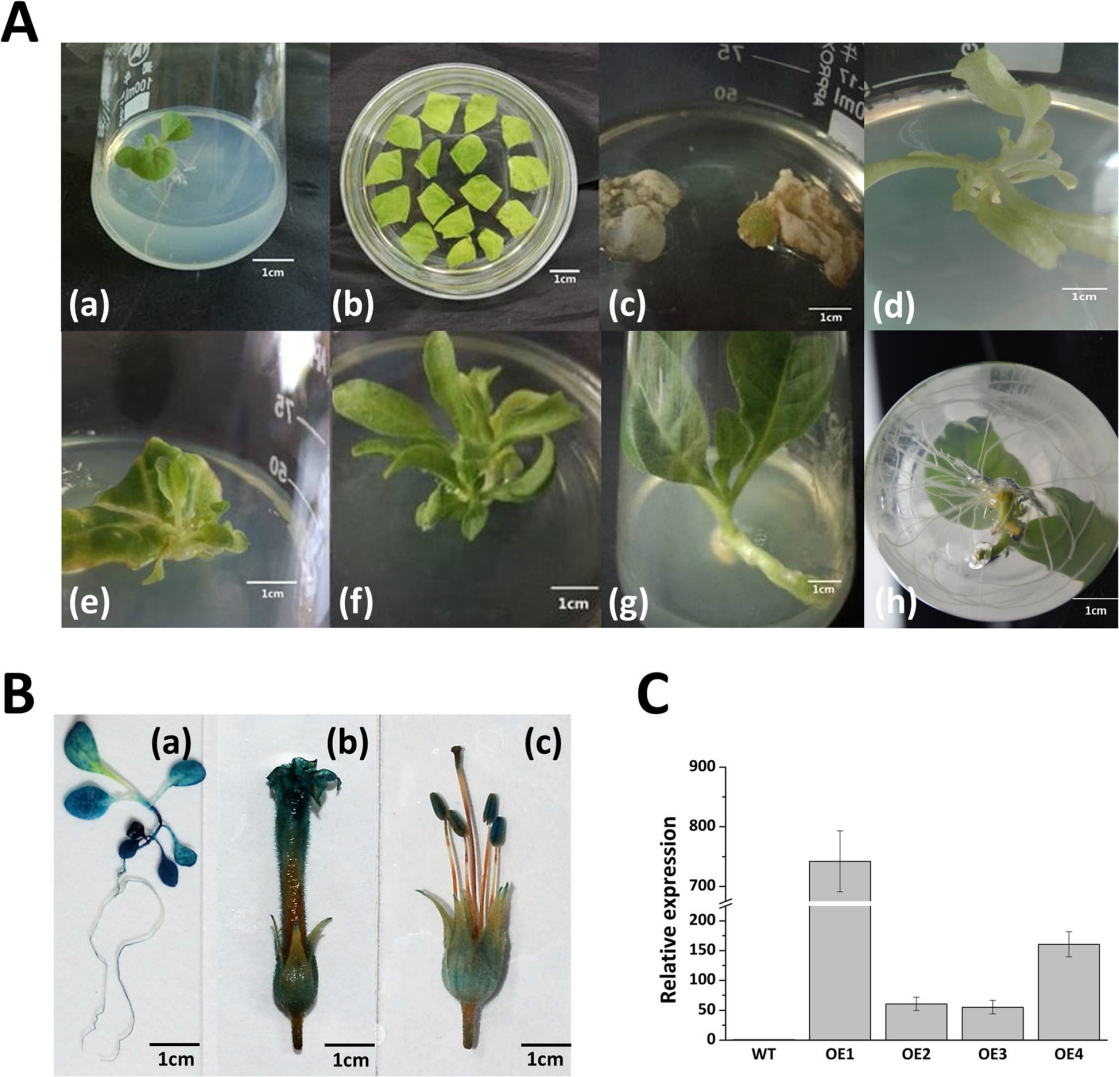
Regeneration of transgenic tobacco.A: (**a**) Tobacco plantlets up to 4–5 leaves were selected for infection; (**b**) Tobacco leaves infected with *Agrobacterium tumefaciens* were cultured in a sterilized culture medium; (**c** and **e**) Budding of tobacco leaves after induction in budding medium; (**d**) Tobacco that did not transform successfully after being kanamycin-screened died of whiteness; (**f**-**h**) Growth of tobacco roots in rooting medium; (**b**) GUS staining of transgenic tobacco seedlings, pistils, and stamens; (**c**) Transcript levels of *Fm4CL-like 1* in different overexpression lines.

According to the expression analysis of the *Fm4CL* gene family in *Fraxinus mandshurica*, *Fm4CL-like 1* was chosen for overexpression in transgenic tobacco. *Agrobacterium* strain LBA4404 containing the pBI121- *Fm4CL-like 1* recombinant plasmid was used to mediate the genetic transformation of tobacco leaves. The transformed tobacco was placed directly in differentiation-selective MS medium (with 50 mg/L NAA and 500 mg/L 6-BA) containing 50 mg/L kanamycin and 300 mg/L cefotaxime. The above-mentioned tobacco leaves were differentiated into shoots on differentiation media to obtain kanamycin-resistant tobacco plants. Finally, the tobacco plants were rooted in MS medium containing 200 mg/L NAA (Fig. 3a). Then, the expression of *Fm4CL-like 1* in transgenic tobacco was detected by real-time RT-PCR. Ten independent transgenic events were obtained. *Fm4CL-like 1*-overexpressed transgenic line 1 was selected for further study based on the expression level.

GUS staining of transgenic plants showed that the GUS activity in the young leaves and stems was the highest among tissues in the three-week-old transgenic tobacco seedlings, while the activities in the root and mature petiole were lower. GUS expression was also detected in transgenic tobacco flower tissues. Among these, the GUS activity was highest in the petals, anthers, receptacle, calyx, and stigma. However, the filaments and pedicel hardly exhibit GUS activity. The above results are consistent with the results of real-time RT-PCR, demonstrating that *Fm4CL-like 1* is highly expressed in transgenic tobacco (Fig. 3c).

### *Fm4CL-like 1* plays an important role in lignin biosynthesis

**Fig. 4:**
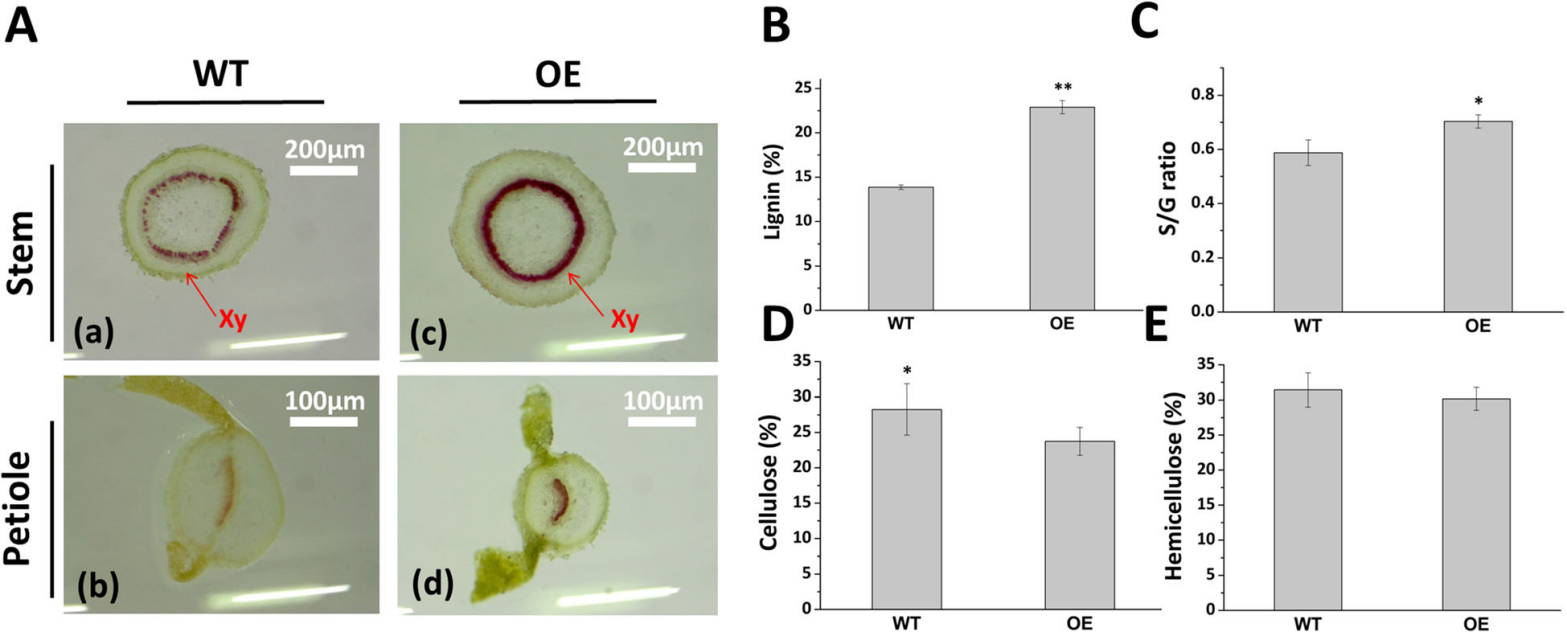
Cell wall component analysis (**a**) Sections stained with phloroglucinol-HCl in WT (**a**, **b**) and OE (**c**, **d**) plants; Xy, xylem. (**b**-**e**) Secondary cell wall composition of WT and OE plants, including lignin, S/G lignin ratio, cellulose, and hemicellulose. Data were represented as the mean ± SE of three biological replicates; asterisks indicate levels of significance (t-test; ∗ *P* < 0.05, ∗∗ *P* < 0.01).

To understand the function of *Fm4CL-like 1*, the contents of lignin, cellulose, and hemicellulose were measured in transgenic tobacco. The lignin content in overexpression lines (OE) was approximately 39.5% higher than that in WT (*P* < 0.05) (Fig. 4b), and the S/G ratio of transgenic tobacco increased by 19.7% compared with WT (*P* < 0.05) (Fig. 4c). The cellulose content in WT was approximately 14.78% higher than that in OE lines (*P* < 0.05) (Fig. 4d). The hemicellulose content was very similar in both conditions (Fig. 4e). In addition, the stems and petioles of OE line were sectioned and stained with phloroglucinol-HCl (Fig. 4a). Based on xylem staining, the lignin content of OE plants was higher than that of WT, indicating that *Fm4CL-like 1* promoted lignin deposition. These results demonstrate that overexpression of *Fm4CL-like 1* contributes to the accumulation of lignin in tobacco.

### *Fm4CL-like 1* altered the xylem of transgenic tobacco

**Fig. 5:**
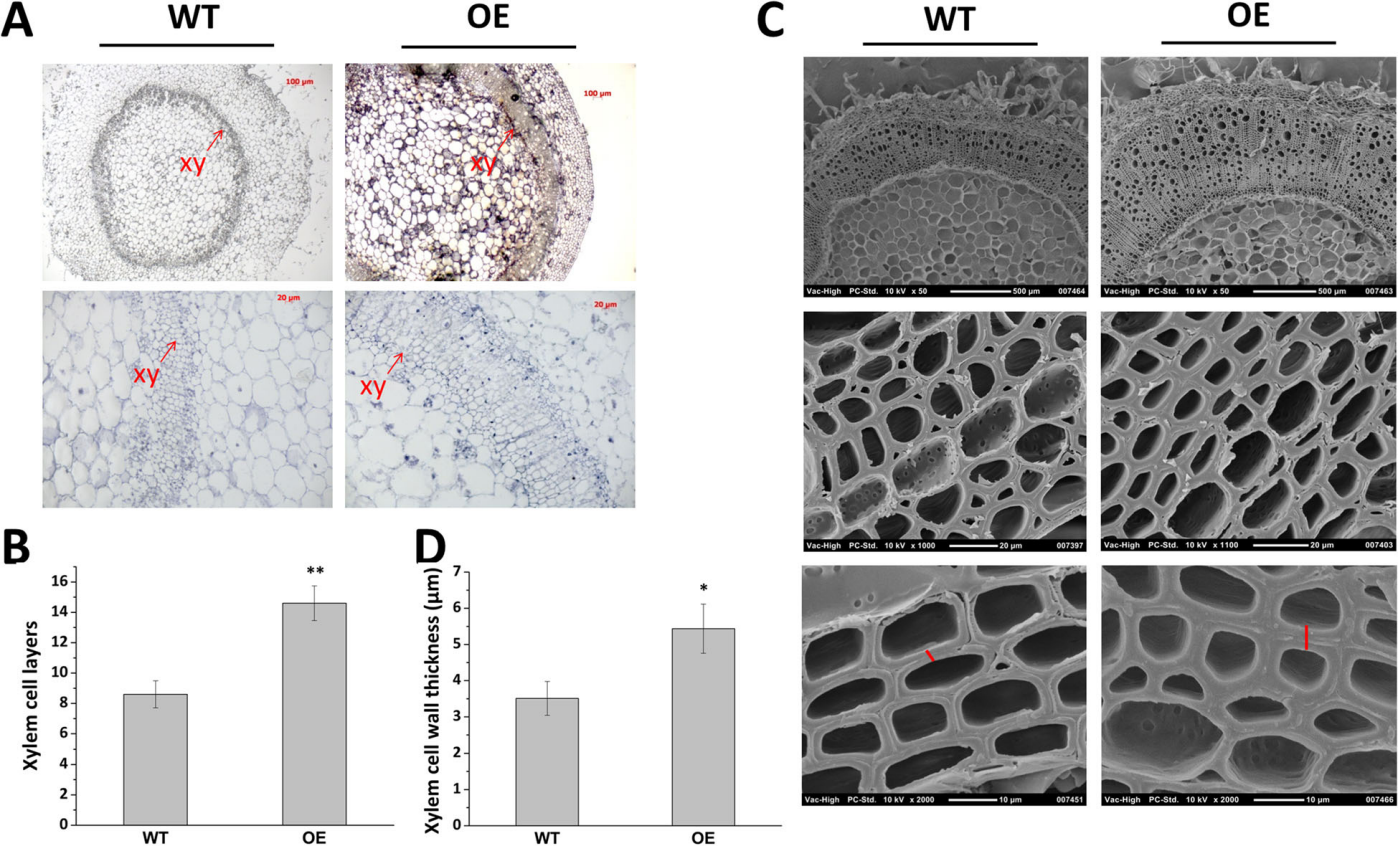
Microstructure of xylem cells (**a**) Paraffin section pictures of WT and OE stem segments. (**b**) Xylem cell layers of WT and OE. (**c**) Sections were analyzed by scanning electron microscopy for WT and OE. (**d**) Secondary wall thickness of xylem fibers of WT and OE. The sections were analyzed from the same stem positions of different plants of the same transgenic line. Data were represented as the mean ± SE of three biological replicates; asterisks indicate levels of significance (t-test; ∗ *P* < 0.05, ∗∗ *P* < 0.01).

The third section of OE and WT was paraffin-embedded (Fig. 5a) and we found that the number of xylem cells in OE lines increased by 40% compared with WT plants (Fig. 5b). Xylem cell walls of OE and WT were observed by scanning electron microscopy (Fig. 5c). The cell wall thickness of WT and OE samples was respectively about 1.91 ± 0.13 μm and 2.43 ± 0.08 μm, indicating that the cell wall thickness of OE was increased by 21.6% compared to WT (Fig. 5d). This is the further evidence that *Fm4CL-like 1* is involved in the synthesis of plant xylem.

### *Fm4CL-like 1* confers mannitol tolerance

**Fig. 6:**
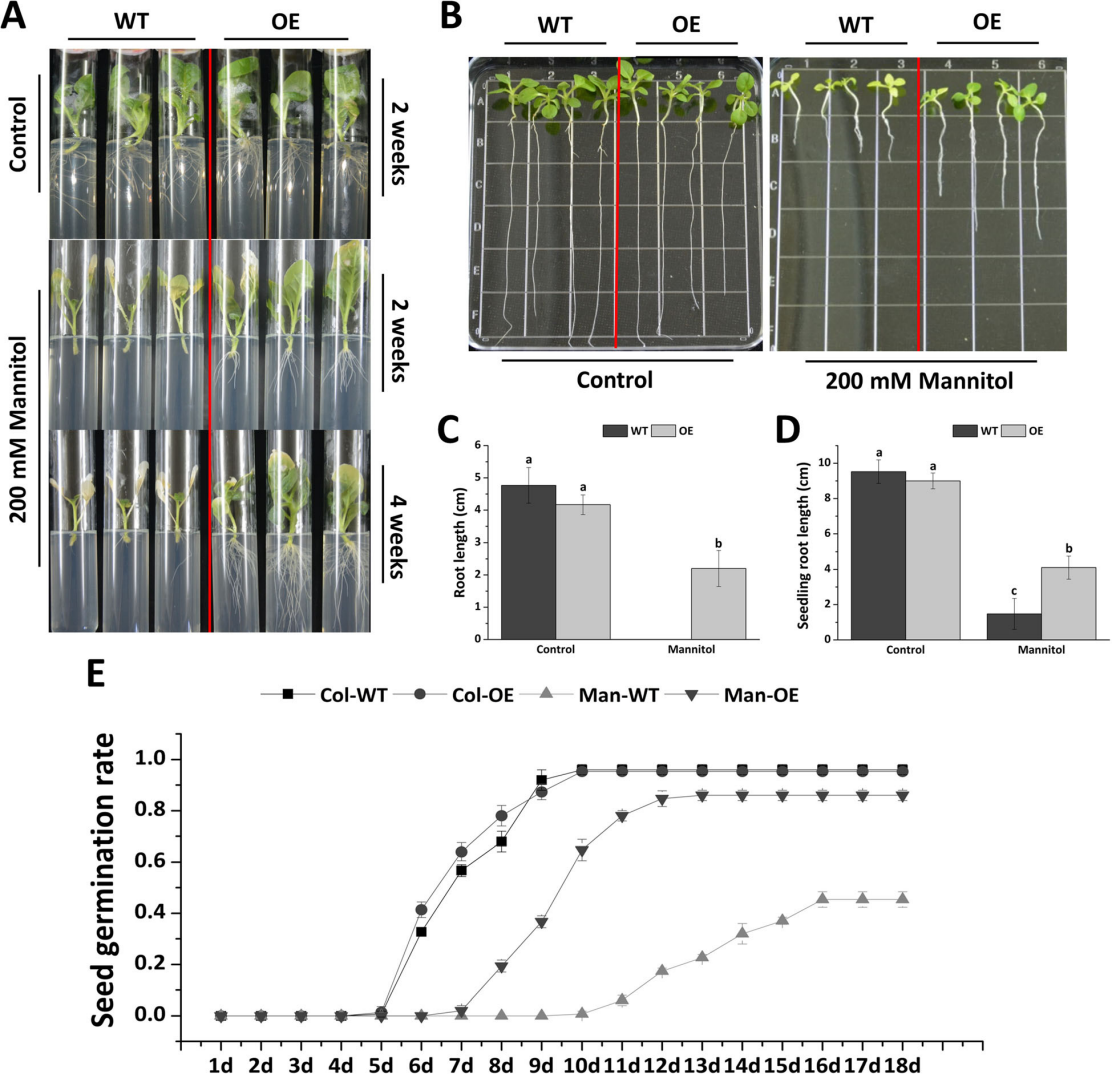
*Fm4CL-like 1* increases the mannitol stress tolerance of transgenic tobacco lines (**a**, **b**) Comparison of growth phenotypes of WT and OE tobacco plants under control and mannitol stress conditions. (**c**) Root length of WT and OE tobacco plants under control and mannitol stress conditions. (**d**) Root length of WT and OE tobacco T1 seedlings under control and mannitol stress conditions. (E) Germination of WT and OE seeds under control and mannitol stress conditions. Data were represented as the mean ± SE of three biological replicates; statistical significance (*P* < 0.05) between WT and OE is indicated by **a**, **b**, and **c**.

Transgenic and WT tobacco plants were treated with 200 mM Mannitol to study their tolerance to osmotic stress. Under the control conditions, no significant difference in the phenotype, growth rate, fresh weight, or root length of WT and OE samples was noted (Fig. 6a), indicating that *Fm4CL-like 1* did not affect the phenotype or growth rate of the plant.

After two and four weeks of mannitol treatment, OE showed a higher growth rate, greener leaves compared to WT plants. After four weeks treatment, root length of OE is 64% longer than WT (Fig. 6a and c). Treatment of WT and OE seedlings with 200 mM Mannitol, the result was similar, OE seedlings have a longer root and greener leaves than WT, while WT plants were grown slowly and the leaves turned yellow (Fig. 6b and d).

The seed germination rate of WT and OE lines grown in normal MS medium were similar. However, with 200 mM mannitol treatment, the seed germination rate of OE is 47% higher than that of WT (Fig. 6e). Moreover, OE seedlings began to germinate on the 4th day after sowing, while WT seedlings began to germinate on the 6th day. These results suggest that overexpression of *Fm4CL-like 1* significantly increased osmotic stress tolerance in tobacco.

### *Fm4CL-like 1* affects the accumulation of reactive oxygen species (ROS), biosynthesis of MDA, and relative conductivity

**Fig. 7:**
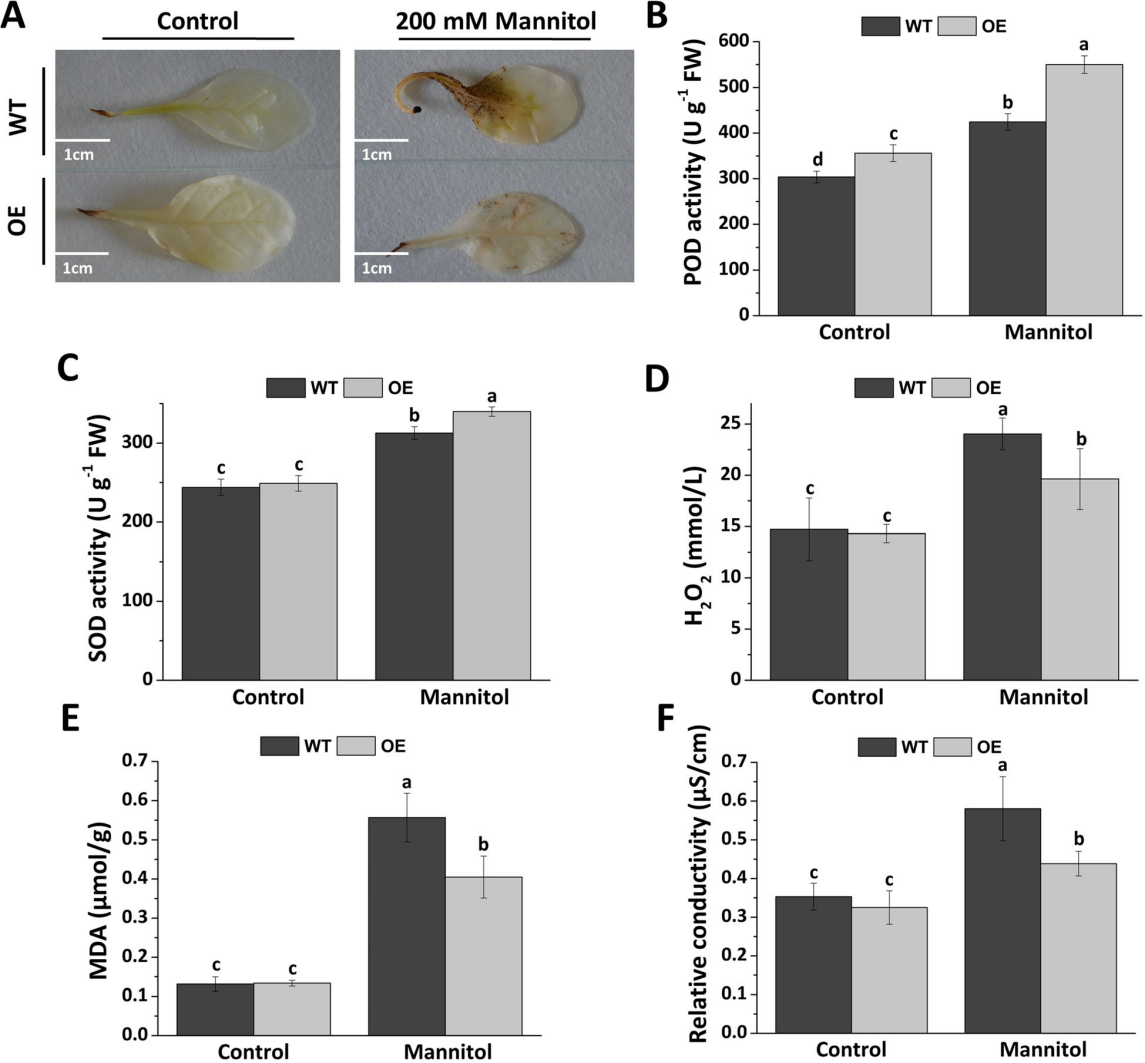
Detection of ROS accumulation of WT and OE plants under control and mannitol stress conditions. (**a**) Detection of ROS using DAB in situ staining (**b**) POD activity (**c**) SOD activity (**d**) Hydrogen peroxide content (**e**) MDA activity (F) Conductivity activity; Data were represented as the mean ± SE of three biological replicates; statistical significance (*P* < 0.05) between mannitol treatments is indicated by **a**, **b**, and **c**

ROS is a plant signaling molecule. Under drought stress, plant cells will produce a large amount of ROS resulting in inhibition of plant growth [39]. Therefore, we investigated whether *Fm4CL-like 1* could affect ROS accumulation. DAB staining was used to assess the level of H_2_O_2_ (a major ROS) (Fig. 7a). No significant difference in DAB staining was observed between WT and OE lines under control conditions. However, OE showed reduced DAB staining compared to WT plants after 3rd d of treatment with 200 mM Mannitol, indicating that *Fm4CL-like 1* overexpression reduced H_2_O_2_ accumulation in plants. We further measured the H_2_O_2_ level (Fig. 7d). Consistent with DAB staining, there was no difference between WT and OE under control conditions. However, with 200 mM mannitol treatment, H_2_O_2_ levels were 22% higher in WT than OE. These results show that *Fm4CL-like 1* can affect ROS accumulation.

Due to the obvious differences in H_2_O_2_ levels in WT and OE lines after mannitol treatment, the activities of POD and SOD were measured in mannitol-treated tobacco. The results showed that the POD and SOD activity in the OE line was higher by 35 and 24% than that in WT (Fig. 7b, c).

The study measured leaf cell conductivity and MDA content of plants on the 3rd d of growth under normal conditions and 200 mM mannitol treatment, respectively. Under control conditions, the MDA and relative conductivity of WT and OE samples were similar. However, under the mannitol treatment, the MDA level of the WT was 25% higher than that of OE (Fig. 7e). The relative conductivity of the WT was 15% higher than that of OE (Fig. 7f). These results indicate that WT tobacco is more susceptible to damage than transgenic tobacco in a mannitol-simulated arid environment.

### *Fm4CL-like 1* reduces water loss by reducing the stomatal apertures

**Fig. 8:**
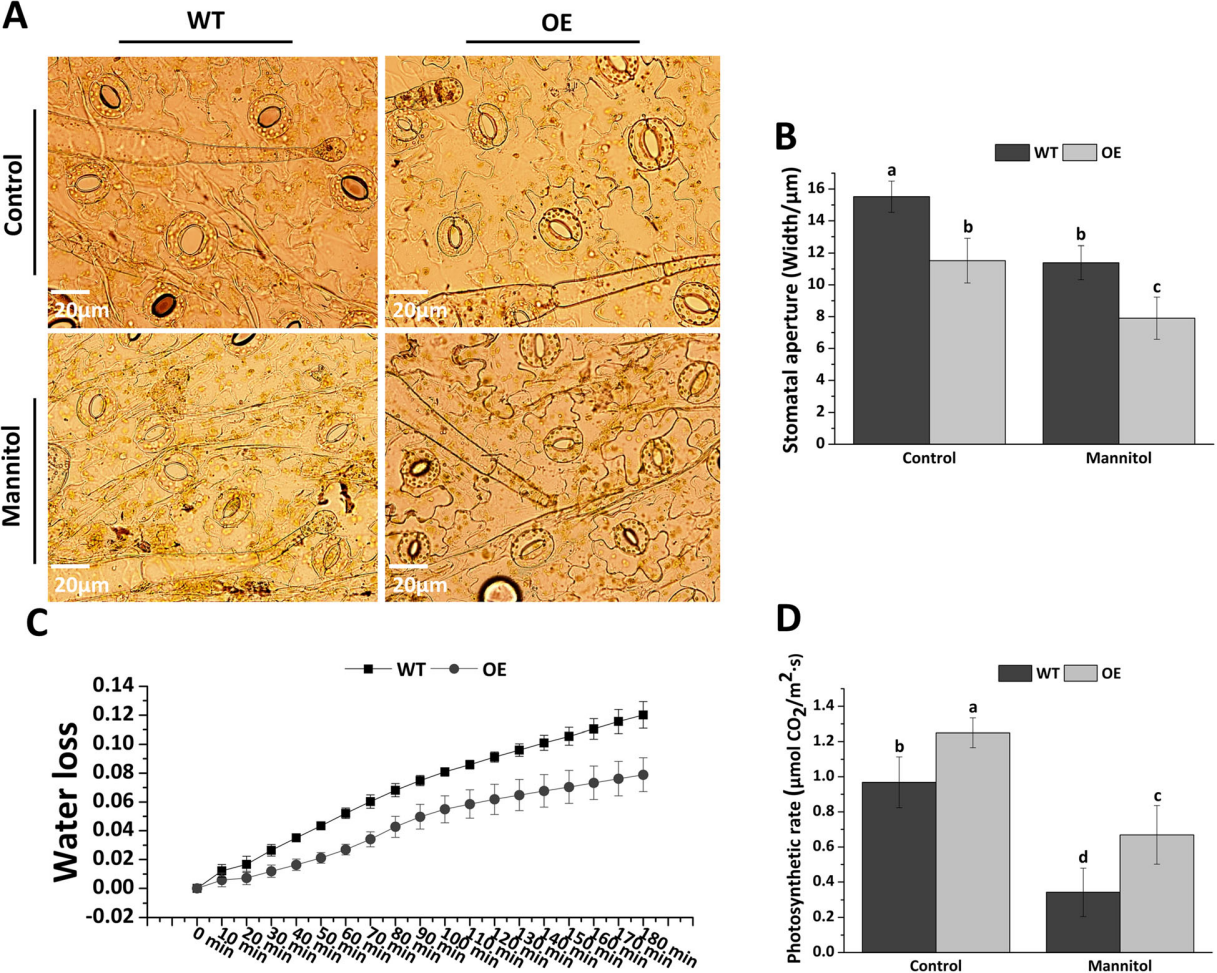
Analysis of stomatal aperture, water loss rate, and photosynthetic in WT and OE tobacco lines. (**a**, **b**) Light microscopic observation of WT and OE lines stomata under control conditions and 200 mM mannitol conditions (**c**) Photosynthetic rates (**d**) Water loss rates. Data were represented as the mean ± SE of three biological replicates; statistical significance (*P* < 0.05) between mannitol treatments is indicated by **a**, **b**, and **c**

Since OE is more resistant to osmotic stress than WT, we suspect that it may be also related to changes in the stomata. The leaf stomata of WT and OE plants were observed by light microscopy. The stomatal apertures of OE were 25% smaller than those of WT under control conditions. Under mannitol stress, the stomatal apertures of both WT and OE were reduced in size, while the stomatal apertures of OE were 30% smaller than those of WT (Fig. 8a-b). The photosynthetic rate of OE was 22% higher than that of WT under the control conditions. However, the photosynthetic rate of OE was 48% higher than that of WT under mannitol stress conditions (Fig. 8c). These results indicate that photosynthesis of plants can be affected by changes in the stomatal structure of the leaves. Since transgenic tobacco plants had a stronger drought tolerance than WT, we measured the water loss rates of WT and transgenic tobacco plants in vitro. The water loss rate of WT plants was 16.7% higher than that of the OE group (Fig. 8d). The higher water loss was detected when the detached leaves exposed to air, indicating that *Fm4CL-like 1* overexpression reduced the plant transpiration rate.

### *Fm4CL-like 1* affect the expression of stress-related genes

**Fig. 9:**
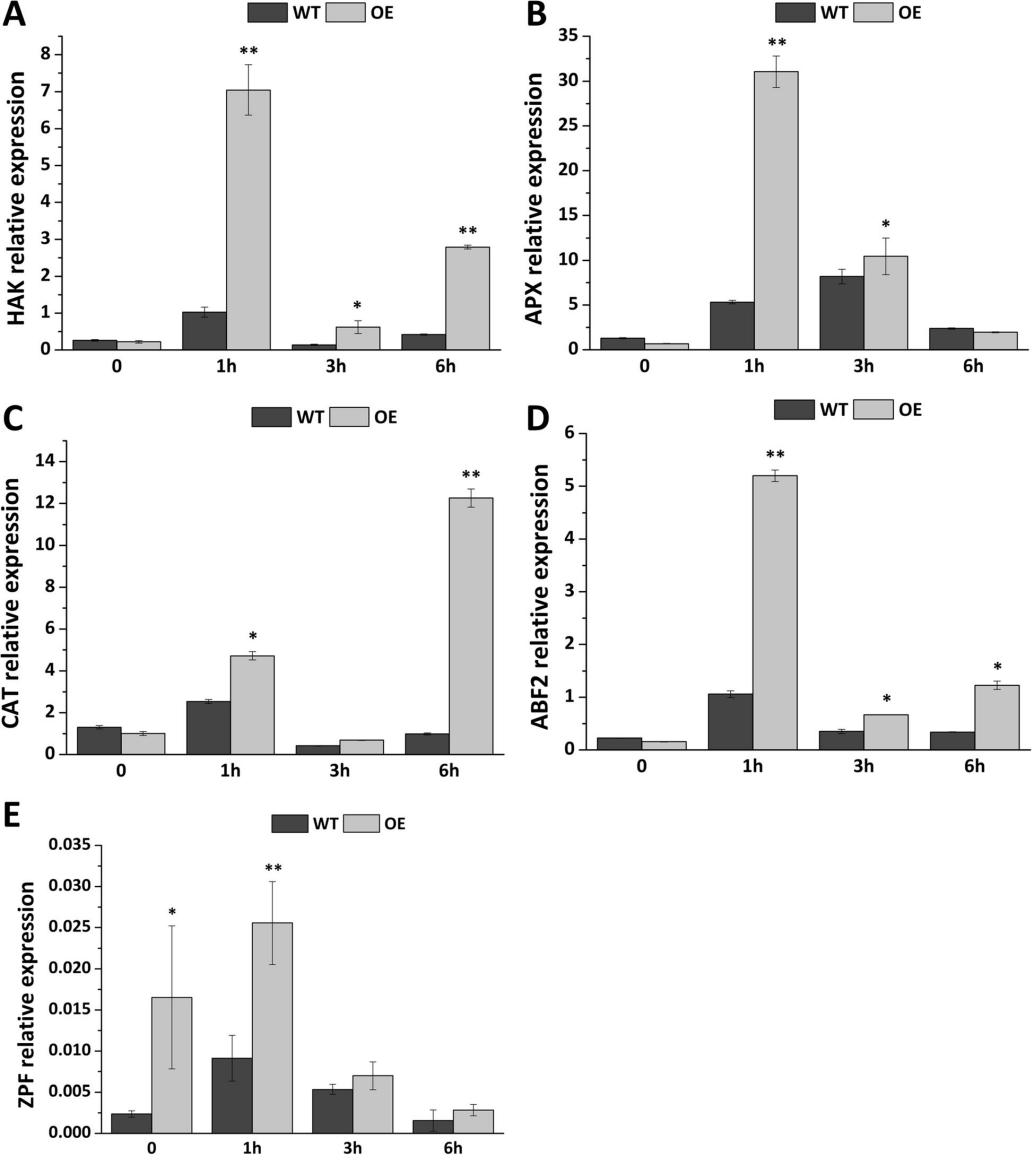
Relative expression of genes involved in stress response in transgenic lines of the WT and OE lines. Data were represented as the mean ± SE of three biological replicates; asterisks indicate levels of significance (t-test; ∗ *P* < 0.05, ∗∗ *P* < 0.01)

From the previous determination of physiological indicators (e.g., ROS), five stress-related response genes were selected for the quantification of expression levels, to analyze the role of *Fm4CL-like 1* in anti-retrograde pathway at the molecular level (Fig. 9). The results showed that the transcriptional expression of *NtABF2*, *NTZFP*, *NTCAT*, *NtHAK1*, and *NTAPX* genes were significantly different between WT and OE samples. Under normal growth conditions, the expression levels of *NtABF2*, *NtCAT*, *NtAPX*, and *NtHAK1* in WT and OE were not significantly different, while the expression level of *NtZFP* in OE was 7 times higher than in WT. After mannitol treatment, these five genes were up regulated in WT and OE. The expression of *NTHAK1*, *NTABF2*, *NTZFP*, and *NTAPX* was at the peak after 1 h treatment. The expression level of *NtCAT* peaked 6 h after mannitol treatment. The expression levels of these five genes in OE after mannitol treatment were higher than those in WT. These results indicate that *Fm4CL-like 1* can affect stress response gene expression.

## Discussion

Research on lignin biosynthesis pathway-related enzymes is gaining attention. 4CL is a plant phenylpropane derivative, which is a key enzyme in the lignin biosynthesis pathway [40]. In this study, 12 *4CL* genes were obtained from the *Fraxinus mandshurica* transcriptome. To identify the function of Fm4CL proteins, we predicted and analyzed the Fm4CL protein structure using online bioinformatics analysis tools. The prediction analysis showed that *Fm4CL2*, *Fm4CL3*, and *Fm4CL11* were unstable proteins and the remaining were stable proteins (Additional file 1: Table S1). If the total predicted average hydrophobicity is normal, this indicates that the protein is a hydrophobic protein. If it is outside normal values, the protein is hydrophilic. Our results showed that *Fm4CL4* (*Fm4CL-like 1*), *Fm4CL1*, *Fm4CL1*, *Fm4CL5*, and *Fm4CL6* were hydrophilic proteins and the rest were hydrophobic proteins. The Fm4CL protein has a transmembrane domain, indicating that the Fm4CL protein has transmembrane capabilities. The secondary structure of the Fm4CL protein was analyzed using GOR4 [41]. The results showed that all the *Fm4CL* proteins were composed of alpha helix, extended strand, and random coil domains, distributed throughout the protein.

Studies have shown that the tobacco *4CL* is activated by injury [42]. Tension treatment of the wood clearly demonstrated the reduction of wood lignin deposition using lignin staining in poplar after mechanical bending for 6 h [43]. However, this decrease was transient as the transcription level gradually approached the control after 1 d [44]. In *Populus* species, TW had a lignin content 20% lower than that of untreated wood after one week of tension [45]. This is similar to our result (Fig. 2b), in which the expression of *Fm4CL* was similar to that of control treated for 1 d. The expression level of *Fm4CL* in the sample treated for 7 d was lower than that of the control. The results of Gui [28] and Voelker [46] indicate that the effect on lignin reduction of plant growth may depend on the severity of the environmental conditions, and decrease in lignin content leads to a decrease in the strength and stiffness of the wood and an increase in the incidence of the wood.

Hu [18] applied antisense technology to *Pt4CL1* from *Populus euphratica* to analyze the regulation of poplar lignin synthesis. The lignin of transgenic poplars that inhibited the expression of *Pt4CL1* decreased by up to 45%. However, this loss was offset by the 15% increase in cellulose, indicating that lignin and cellulose deposition could be controlled by a compensation mechanism [18]. Kajita generated transgenic tobacco with *4CL* expression inhibition [47]. Lignin content decreased by 19 to 35%, cellulose content increased, but the total content of lignin and cellulose unchanged compared with the WT [47]. Our result are similar (Fig. 4b, d, e), with increased lignin content and reduced cellulose content in transgenic tobacco, suggesting that the synthesis of lignin and cellulose in plants can compensate for each other. Compared with WT, the S/G ratio in OE increased by 19.7%, which indicated that *Fm4CL-like 1* plays an important role in S-lignin subunit synthesis pathway. The increase in S/G ratio accelerated the maturation of secondary xylem cells [48], This is consistent with the microscopic observations of our study. By microscopic observation of the paraffin sections of the WT and OE stem segments, it was clearly observed that the transgenic tobacco was more lignified, with more layers of xylem cells and thicker cell walls than WT tobacco (Fig. 5a-d). This increase may enhance the plant’s resistance to stress.

The molecular mechanisms of plants tolerating stress is complicated. Several hundred genes have now been identified in plants that respond to plant biotic or abiotic stresses. Many studies report genes related to stress responses, including genes involved in the biosynthesis of protective osmolytes or transgenes into plants to improve stress tolerance. The hydrophobicity of lignin is thought to have an inhibitory effect on the transpiration of plant tissue under drought conditions [49]. Li [50] reported that loss of *CLD1* / *SRL1* function in rice reduced the lignin content and led to a reduction in drought resistance, further demonstrating the role of lignin in drought resistance. Since lignin in OE was increased by 39% compared to WT, we hypothesized that the OE strain might be resistant to drought and conducted a mannitol-simulated drought treatment. The results showed that the resistance of transgenic tobacco to stress was significantly higher than WT, which indicated that *Fm4CL-like 1* could improve plant drought tolerance.

Under stress, plants produce large quantities of hydrogen peroxide. POD and SOD can remove hydrogen peroxide and POD can convert carbohydrates in the tissues into lignin to increase the degree of lignification [51] and reduce cell damage [52]. We performed DAB staining and quantification of hydrogen peroxide to analyze reactive oxygen species in plants. Compared with OE, WT tobacco accumulated more reactive oxygen under the mannitol treatment, and OE had higher POD and SOD activity (Fig. 7a-d). Under sterss conditions, redox levels in plant cells are out of balance, resulting in the destruction of plant cell membranes. Changes in relative conductivity can reflect the extent of the damage of the cell membrane. In this study, the relative electrical conductivity of transgenic tobacco was lower than that of WT tobacco after treatment with mannitol (Fig. 7e-f), indicating that the *Fm4CL-like 1* is involved in maintaining the integrity of the transgenic tobacco cell membrane and improving the drought resistance. Stomata are the channels for the exchange gasses in photosynthesis. The size of stomatal openings affects the transpiration and photosynthesis of plants [53]. Our results suggest that *Fm4CL-like 1* may increase plant resistance to osmotic stress by decreasing stomatal aperture. Under osmotic stress conditions, the photosynthesis rate of OE was 48% higher than that of WT, suggesting that *Fm4CL-like 1* plants may accumulate more biomass through enhanced photosynthesis to increase tolerance to drought stress (Fig. 8).

After mannitol treatment, WT and OE had distinct changes in the antioxidant enzyme activity and other physiological indicators, indicating that OE is likely to have further changes at the molecular level. Therefore, we chose five stress response genes to determine the expression level. *ABF* is an ABRE binding factor that specifically binds to the G-box and ACGT in the ABRE cis-acting element, resulting in the expression of the drought-responsive response gene *RD29B* [54, 55]. Zinc finger proteins (*ZFPs*) play an important role in the abiotic stress response in plants and can be upregulated under stress conditions [56]. *CAT* and *APX* are key enzymes that scavenge free radicals in plants. They can produce O^2−^, H_2_O_2_, and other reactive oxygen species in plant cells under stress and the stress resistance of the plant is related to *CAT* and *APX* activities. The results showed that the expressions of *NtABF2*, *NtZFP*, *NtCAT*, and *NtAPX* in OE were higher than those of WT plants after mannitol treatment (Fig. 9), which further indicated that *Fm4CL-like 1* may affect the endurance of OE at the molecular level.

These results indicate that overexpression of *Fm4CL-like 1* can promote lignin biosynthesis and lead to thickening of the xylem cell wall in *Fm4CL-like 1* transgenic tobacco, and *Fm4CL-like 1* transgenic tobacco has improved drought resistance compared to WT. Our study shows that the anti-retroactive function of *Fm4CL-like 1* may be related to the stomatal aperture, although this hypothesis is currently not supported by relevant literature and further studies are needed.

## Methods

### Plant materials

Leaves, xylem, bark, buds, flower, bud, petiole, and stem of *Fraxinus mandschurica* were collected from the experimental forestry farm of Northeast Forestry University (Harbin, China) in May, 2013. Five-year-old *Fraxinus mandshurica* specimens were selected for artificial bending in July. Trunks of 1.0–1.5 m were bent to an angle of 45° from vertical. The upper part was parallel to the ground and the directions of inclination were all the same. Samples were collected on the 1st, 3rd, and 7th d of treatment, with 3 replicates at each time point. Untreated *Fraxinus mandshurica* specimens were used as a control, which were collected from the xylem of Tension Wood (TW) and Opposite Wood (OW). After freezing in liquid nitrogen treatment, samples were stored at − 80 °C refrigerator until use. The *Nicotiana tabacum* cultivar Longjiang 911 was used as the WT tobacco strain in this study.

### Total RNA extraction and real-time PCR analysis

Total RNA was extracted from the plant material by the CTAB method with the modifications described by Zeng et al. [57]. First-strand cDNA was synthesized using the Takara™ first strand cDNA synthesis kit (Takara, Dalian, China). The cDNA was quantified by real-time PCR using a 20 μL reaction system using SYBR Premix Ex Taq TM (TaKaRa, Dalian, China) on an ABI Step One Plus system. The real-time qRT-PCR conditions were as follows: 5 min at 95 °C for denaturation, followed by 45 cycles of 8 s at 95 °C, 30 s at 58 °C, and 20 s at 72 °C for amplification. The data were normalized using the reference gene *Fraxinus mandshurica α-tublin* [58] and *Nicotiana tabacum Actin* [59], Gene-specific primers for qRT-PCR analysis were listed in Additional file 1: Table S2. Three biological replicates were used per treatment or control. Raw data of relative quantification values were calculated using the 2^−ΔΔCT^ algorithm approach. Data was processed using the IBM SPSS statistics 19 software, drawing software and USES Origin 8.5 software were used for data analyses and drawing, respectively. All data were repeated three times.

### Isolation of full-length *Fm4CL-like 1* cDNA

The full-length of *Fm4CL-like 1* cDNA was isolated by RT-PCR from the leaves of *Fraxinus mandschurica* using the cDNA F/R primers in Additional file 1: Table S2. Homologous sequences were identified from the National Center for Biotechnology Information (NCBI) database using the BLAST method with the full-length *Fm4CL-like1* cDNA sequence. To generate a phylogenetic tree, DNAMAN software was used to perform the multisequence alignment. Phylogenetic and molecular evolutionary analyses were conducted using the MEGA 6.0 program with the neighbor-joining (NJ) method.

### Generation of the OE line transgenic plants and GUS staining

Full-length cDNA of *Fm4CL-like 1* of *Fraxinus mandshurica* (deposited in GenBank with accession number KJ531403) was amplified using the primers listed in Additional file 1: Table S2, which has a full-length of 1994 bp and an ORF of 1572 bp encoding 523 amino acids. The sequence was then cloned into pBI121, a plant expression vector containing kanamycin-resistant genes driven by the cauliflower mosaic virus (CaMV) -35 s promoter (35S, *Fm4CL-like 1*) using the In-Fusion method [60]. The recombinant vector was introduced into the *Agrobacterium* strain LBA4404 and transformed into WT tobacco using tri-parental mating. Transgenic tobacco plants were regenerated from transformed leaves on selection Murashige and Skoog medium containing 50 mg/L kanamycin and 300 mg/L cefotaxime. *Fm4CL-like 1* levels in the transgenic tobacco plants were further confirmed by real-time PCR. Transgenic tobacco seedlings and flowers were collected for GUS staining and decolorization after taking pictures.

### Drought stress treatment of tobacco

Transgenic and WT tobacco plants with the same growth status in tissue culture were selected and transplanted into MS medium containing 200 mM mannitol to observe their phenotypic changes, including measurement of root length changes. For seed germination assays, seeds of WT and transgenic tobacco were grown on MS medium containing 200 mM Mannitol and grown under prolonged light (16 h light/8 h dark) at 22 °C and observed every 12 h of its germination, until the germination rate was constant.

### Histological analysis of tobacco stem segments

Stem segments of two-month-old transgenic and WT tobacco plants were collected and fixed in FAA (Formaldehyde-Acetic acid-Alcohol solution) solution for 2 d, then stained with hematoxylin for 3 d, and the water was blued for 3 d. Then the samples were dehydrated, waxed, embedded, sliced, stuck to a sheet, and coverslipped for microscope analysis and photography [61]. Sliced dewaxed stem segments were also used to visualize xylem cell walls under scanning electron microscopy. Samples from the top one/third part of the stem of two-month-old transgenic or WT tobacco plants were soaked in a drop of 1 M hydrochloric acid soaked material for 3 min, followed by a drop of 10% phloroglucinol ethanol-hydrochloric solution [62], and visualized quickly under the optical microscope for photographing. For scanning electron microscopy (SEM), The third inter-segment stem segment of two-month-old soil grown WT and OE-*Fm4CL-like 1* tobacco plant was dried by a carbon dioxide zero-point dryer (EM CPD300, Leica, Germany) and used for scanning electron microscope observation (S-4800, HITACHI, Tokyo, Japan). The xylem cell wall thickness in the SEM micrographs was measured using Image J software (https://imagej.nih.gov/ij/).

### Cell wall component analysis

One-month-old transgenic and WT tobacco plants were collected, oven dried at 65 °C to constant weight, and mortar ground over an 80-mesh sieve. Lignin content was determined by the acetyl bromide method [63]. Cellulose content was measured by concentrated sulfuric acid hydrolysis [64]. Hemicellulose content was determined by a combination of hydrochloric acid hydrolysis and DNS [65].

### Measurements of stomatal aperture, water loss, and photosynthetic rate

One month old tissue culture samples of WT and OE tobacco leaves under control conditions were sampled and 200 mM mannitol was used to remove the epidermis. The stomatal apertures were observed and photographed using an optical microscope (Leica Microsystems, Germany). Three measurements were taken for each sample. To determine the water loss rate of plants, 8-week-old WT and OE transgenic plants were cut and weighed immediately (fresh weight, FW) and then dried naturally. Weights of the drying leaves (DW) were taken every 10 min and water loss was calculated using the formula (FW–DW) / FW. The photosynthetic rate was measured using the Li-6400XT Portable Photosynthesis System (Li-COR) according to the manufacturer’s instructions.

### Measurements of MDA content and relative conductivity

One-month-old *Fm4CL-like 1* transgenic and WT tobacco plantlets were grown in MS medium containing 200 mM mannitol for 48 h and used for the determination of the physiological index. MDA content was determined using the thiobarbital acid (TBA) colorimetric method, based on that described by Meir et al. [66]. Approximately 0.5 g leaves were homogenized with 2 mL of 0.1% trichloroacetic acid (TCA) and centrifuged at 8000×g for 10 min. After mixing 2 mL of supernatant with 0.67% TBA, the mixture was incubated in hot water for 15 min, cooled immediately on ice, and centrifuged at 4000×g for 10 min. Absorbances at 532, 450, and 600 nm were determined and MDA concentration was estimated by subtracting the non-specific absorptions at 450 and 600 nm from the absorption at 532 nm; To measure comparative electrical conductivity, 0.5 g leaves were washed with tap water, rinsed 3 times with distilled water, surface moisture removed by blotting with filter paper. The leaves were cut into strips of suitable length, placed in 10 ml of deionized water, and soaked at room temperature for 12 h. The conductivity of the leachate (R1) was measured with a conductivity meter and heated in a boiling water bath for 30 min, cooled to room temperature, and shaken again to determine the conductance of the leachate (R2) relative conductivity = R1 / R2 × 100%.

### Measurement of H_2_O_2_, SOD, and POD content in fresh leaves and DAB staining

To detect the generation of hydrogen peroxide due to abiotic stress, one-month-old *Fm4CL-like 1* transgenic and WT tobacco plantlets were grown in MS medium containing 200 mM mannitol for 48 h. Hydrogen peroxide content was quantified using a hydrogen peroxide kit (Suzhou Keming biotechnology company). Treated tobacco leaves were stained with DAB and stained overnight for 16 h. After staining, leaves were decolorized with 100% ethanol and photographed once the chlorophyll had been completely removed. The SOD activity was determined by the nitrogen blue tetrazolium photoreduction method [67]. The guaiacol method was used to determine the POD activity.

## Additional file

Additional file 1: **Table S1.** Bioinformatics Analysis of *Fm4CL/4CL-like* genes in *Fraxinus mandshurica.* **Table S2.** Primers used for PCR and qRT-PCR assays. (DOCX 23 kb)

## Abbreviations

4CL: 4-Coumarate:CoA ligase
6-BA: 6-Benzylaminopurine
ABF: Aber binding factor
APX: Ascorbate peroxidase
CAT: Catalase
CTAB: Cetyltrimethyl Ammonium Bromide
DAB: 3-diaminobezidin
GUS: β-Galactosidase
HAK: High-affinity K^+^
MDA: Malondialdehyde
MS: MS culture medium
NAA: 1-naphthylacetic acid
ORF: Open reading frame
OW: Opposite wood
POD: Peroxidase
qRT-PCR: quantitative reverse transcription polymerase chain reaction
ROS: Reactive oxygen species
SOD: Superoxide dismutase
TW: Tension wood
ZFP: Zinc finger protein

## References

[1] Penning B, Hunter C, Tayengwa R (2009). Genetic resources for maize cell wall biology. Plant Physiol.

[2] Zhong R, Rd M, Himmelsbach D (2000). Essential role of caffeoyl coenzyme a O-methyltransferase in lignin biosynthesis in woody poplar plants. Plant Physiol.

[3] Higuchi T. Biosynthesis and biodegradation of wood components. Academic Press. 1985, pp141–60.

[4] Vance C, And TK, Sherwood R (2003). Lignification as a mechanism of disease resistance. Annu Rev Phytopathol.

[5] Boerjan W, Ralph J, Baucher M. Lignin biosynthesis - Annual Review of Plant Biology. 54(1): 519. 10.1146/annurev.arplant.54.031902.134938 14503002

[6] Campbell M, Sederoff R (1996). Variation in lignin content and composition (mechanisms of control and implications for the genetic improvement of plants). Plant Physiol.

[7] Lin C, Wang J, Li Q (2015). 4-Coumaroyl and caffeoyl shikimic acids inhibit 4-coumaric acid:coenzyme a ligases and modulate metabolic flux for 3-hydroxylation in monolignol biosynthesis of *Populus trichocarpa*. Mol Plant.

[8] Douglas C (1996). Phenylpropanoid metabolism and lignin biosynthesis: from weeds to trees. Trends in Plant Sci.

[9] Hu Y, Li WC, Xu YQ (2009). Differential expression of candidate genes for lignin biosynthesis under drought stress in maize leaves. J Appl Genet.

[10] Geng D, Chen P, Shen X (2018). MdMYB88 and MdMYB124 enhance drought tolerance by modulating root vessels and cell walls in apple. Plant Physiol.

[11] Baucher M, Monties B, Montagu M (1998). Biosynthesis and genetic engineering of lignin. Crit Rev Plant Sci.

[12] Whetten R, Sederoff R (1995). Lignin biosynthesis. Plant Cell.

[13] Whetten R, Mackay J, Sederoff R (1998). Recent advances in understanding lignin biosynthesis. Annu Rev Plant Physiol Plant Mol Biol.

[14] Boudet A (1998). A new view of lignification. Trends Plant Sci.

[15] Boudet A (2000). Lignins and lignification: selected issues. Plant Physiol Bioch.

[16] Winkel-Shirley B (2001). Flavonoid biosynthesis. A colorful model for genetics, biochemistry, cell biology, and biotechnology. Plant Physiol.

[17] Sederoff R, Mackay J, Ralph J (1999). Unexpected variation in lignin. Curr Opin Plant Biol.

[18] Hu W, Harding S, Lung J (1999). Repression of lignin biosynthesis promotes cellulose accumulation andgrowth in transgenic trees. Nat Biotechnol.

[19] Lee D, Meyer K, Chapple C (1997). Antisense suppression of 4-coumarate:coenzyme a ligase activity in *Arabidopsis* leads to altered lignin subunit composition. Plant Cell.

[20] Uhlmann A, Ebel J (1993). Molecular cloning and expression of 4-coumarate:coenzyme a ligase, an enzyme involved in the resistance response of soybean (*Glycine max L*.) against pathogen attack. Plant Physiol.

[21] Hu Y, Gai Y, Yin L (2010). Crystal structures of a Populus tomentosa 4-Coumarate:CoA ligase shed light on its enzymatic mechanisms. Plant Cell.

[22] Dixon R, Achnine L, Kota P (2002). The phenylpropanoid pathway and plant defence-a genomics perspective. Mol Plant Pathol.

[23] Chen H, Babst B, Nyamdari B (2014). Ectopic expression of a loblolly pine class II 4-coumarate: CoA ligase alters soluble phenylpropanoid metabolism but not lignin biosynthesis in *Populus*. Plant Cell Physiol.

[24] Raes J, Rohde A, Christensen J (2003). Genome-wide characterization of the lignification toolbox in *Arabidopsis*. Plant Physiol.

[25] Ehlting J, Büttner D, Wang Q (1999). Three 4-coumarate:coenzyme a ligases in Arabidopsis thaliana represent two evolutionarily divergent classes in angiosperms. Plant J.

[26] Cukovic D, Ehlting J, Vanziffle J (2001). Structure and evolution of 4-coumarate:coenzyme a ligase (4CL) gene families. Biol Chem.

[27] Lindermayr C, Möllers B, Fliegmann J (2002). Divergent members of a soybean (*Glycine max L.*) 4-coumarate:coenzyme a ligase gene family. Eur J Biochem.

[28] Gui J, Shen J, Li L (2011). Functional characterization of evolutionarily divergent 4-Coumarate:coenzyme a ligases in Rice. Plant Physiol.

[29] Hamberger B, Hahlbrock K (2004). The 4-coumarate: CoA ligase gene family in *Arabidopsis thaliana* comprises one rare, sinapate-activating and three commonly occurring isoenzymes. Proc Natl Acad Sci U S A.

[30] Hu W, Kawaoka A, Tsai C (1998). Compartmentalized expression of two structurally and functionally distinct 4-Coumarate:CoA ligase genes in Aspen (*Populus tremuloides*). Proc Natl Acad Sci U S A.

[31] Cardenas C, Cochrane F, Shockey J (2005). Characterization in vitro and in vivo of the putative multigene 4-coumarate: CoA ligase network in *Arabidopsis*: syringyl lignin and sinapate/sinapyl alcohol derivative formation. Phytochemistry..

[32] Zeng F (2014). Drought resistance and DNA methylation of interspecific hybrids between *Fraxinus mandshurica* and *Fraxinus americana*. Trees..

[33] Chen Y, Xue G, Liu F (2016). Immunosuppressive effect of extracts from leaves of *Fraxinus Mandshurica Rupr*. Bioengineered..

[34] Zhang X, Lei L, Lai J (2018). Effects of drought stress and water recovery on physiological responses and gene expression in maize seedlings. BMC Plant Biol.

[35] Asada K, Foyer CH, Mullineaux PM (1994). Causes of photooxidative stress and amlioration of defense systems in plants.

[36] Shereen A, Mumtaz S (2005). Salinity effects on seedling growth and yield components of different inbred rice lines. Pak J Bot.

[37] Khakdan F, Nasiri J, Ranjbar M, Alizadeh H (2017). Water deficit stress fluctuates expression profiles of 4cl, c3h, comt, cvomt and eomt genes involved in the biosynthetic pathway of volatile phenylpropanoids alongside accumulation of methylchavicol and methyleugenol in different iranian cultivars of basil. J Plant Physiol.

[38] Soltani B, Ehlting J, Hamberger B (2006). Multiple cis -regulatory elements regulate distinct and complex patterns of developmental and wound-induced expression of *Arabidopsis thaliana* 4CL, gene family members. Planta..

[39] You J, Chan Z (2015). ROS regulation during abiotic stress responses in crop plants. Front Plant Sci.

[40] Naik P, Wang J, Sederoff R (2018). Assessing the impact of the 4CL enzyme complex on the robustness of monolignol biosynthesis using metabolic pathway analysis. PLoS One.

[41] Sen T, Jernigan R, Garnier J (2005). GOR V server for protein secondary structure prediction. Bioinformatics..

[42] Lee D, Douglas C (1996). Two divergent members of a tobacco 4-Coumarate:coenzyme a ligase (4CL) gene family (cDNA structure, gene inheritance and expression, and properties of recombinant proteins). Plant Physiol.

[43] Jin H, Mi K (2009). Mechanical bending-induced tension wood formation with reduced lignin biosynthesis in *Liriodendron tulipifera*. J Wood Sci.

[44] Paux E, Carocha V, Marques C, Mendes A, Borralho N, Sivadon P (2005). Transcript profiling of eucalyptus xylem genes during tension wood formation. New Phytol.

[45] Andersson-Gunneras S, Mellerowicz E, Love J (2006). Biosynthesis of cellulose-enriched tension wood in *Populus*: global analysis of transcripts and metabolites identifies biochemical and developmental regulators in secondary wall biosynthesis. Plant J.

[46] Voelker L, Lachenbruch B, Meinzer C (2010). Antisense Down-regulation of 4CL expression alters lignification, tree growth, and Saccharification potential of field-grown *Poplar*. Plant Physiol.

[47] Kajita S, Mashino Y, Nishikubo N (1997). Immunological characterization of transgenic tobacco plants with a chimeric gene for 4-coumarate: CoA ligase that have altered lignin in their xylem tissue. Plant Sci.

[48] Li L, Zhou Y, Cheng X (2003). Combinatorial modification of multiple lignin traits in trees through multigene cotransformation [J]. Proc Natl Acad Sci U S A.

[49] Jordan R (1992). Structural changes and associated reduction of hydraulic conductance in roots of *Sorghum bicolor L.* following exposure to water deficit. Plant Physiol.

[50] Li W, Zhang M, Gan P (2017). *CLD1/SRL1* modulates leaf rolling by affecting cell wall formation, epidermis integrity and water homeostasis in rice. Plant J.

[51] Imberty A, Goldberg R, Catesson M (1985). Isolation and characterization of *Populus* isoperoxidases involved in the last step of lignin formation. Planta..

[52] Welinder K (1992). Superfamily of plant, fungal and bacterial peroxidases. Curr Opi Struct Biol.

[53] Azoulay-Shemer T, Palomares A, Bagheri A (2015). Guard cell photosynthesis is critical for stomatal turgor production, yet does not directly mediate CO2-and ABA-induced stomatal closing. Plant J.

[54] Banerjee A, Roychoudhury A (2017). Abscisic-acid-dependent basic leucine zipper (bZIP) transcription factors in plant abiotic stress. Protoplasma..

[55] Kim SY (2006). The role of ABF family bZIP class transcription factors in stress response. Physiol Plant.

[56] Zhang X, Zhang B, Li MJ (2016). *OsMSR15* encoding a rice C2H2-type zinc finger protein confers enhanced drought tolerance in transgenic Arabidopsis. J Plant Biol.

[57] Zeng F, Qian J, Luo W (2010). Stability of transgenes in long-term micropropagation of plants of transgenic birch (*Betula platyphylla*). Biotechnol Lett.

[58] He (2016). Drought physiology and gene expression characteristics of *Fraxinus* interspecific hybrids. Plant Growth Regul.

[59] Schmidt GW (2010). Stable internal reference genes for normalization of real-time RT-PCR in tobacco (*Nicotiana tabacum*) during development and abiotic stress. Mol Gen Genomics.

[60] Decai T (2015). Rapid construction of stable infectious full-length cDNA clone of papaya leaf distortion mosaic virus using in-fusion cloning. Viruses..

[61] Ruzin SE (1999). Plant microtechnique and microscopy.

[62] Pomar F (2002). *O*-4-linked coniferyl and sinapyl aldehydes in lignifying cell walls are the main targets of the Wiesner (phloroglucinol-HCl) reaction. Protoplasma..

[63] Morrison I (1972). A semi-micro method for the determination of lignin and its use in predicting the digestibility of forage crops. J Sci Food Agric.

[64] Thygesen A, Oddershede J, Lilholt H (2005). On the determination of crystallinity and cellulose content in plant fibres. Cellulose.

[65] Foster CE, Martin TM, Pauly M. Comprehensive compositional analysis of plant cell walls (lignocellulosic biomass) part I: lignin. J Vis Exp. 2010;(37). 10.3791/1745 PMC3144576 20224547

[66] Meir S, Philosoph-Hadas S, Gloter P (1992). Nondestructive assessment of chlorophyll content in watercress leaves by a tristimulus reflectance colorimeter. Postharvest Biol & Technol.

[67] Hwang C, Rhie G, Kim S (1999). Copper- and zinc-containing superoxide dismutase and its gene from *Candida albicans*. Biochim Biophys Acta.

