# Supplementary material for: Molecular cloning and functional analysis of 4-Coumarate:CoA ligase 4(4CL-like 1)from Fraxinus mandshurica and its role in abiotic stress tolerance and cell wall synthesis
Source: BMC Plant Biol. 2019 Jun 3;19:231. doi: 10.1186/s12870-019-1812-0 (PMC6545724; doi:10.1186/s12870-019-1812-0)
Supplement: Supplementary file 1 — Table S1. Bioinformatics Analysis of Fm4CL/4CL-like genes in Fraxinus mandshurica. Table S2. Primers used for PCR and qRT-PCR assays. (DOCX 23 kb) [file 12870_2019_1812_MOESM1_ESM.docx]

Table S1: Bioinformatics Analysis of *Fm4CL/4CL-like* genes in *Fraxinus mandshurica*

| **Gene name** | *Fm4CL1* | *Fm4CL2* | *Fm4CL3* | *Fm4CL-like 1* | *Fm4CL5* | *Fm4CL6* |
| --- | --- | --- | --- | --- | --- | --- |
| **Login ID** | KJ531400 | KJ531401 | KJ531402 | KJ531403 | KJ531404 | KJ994781 |
| **Length (bp)** | 2400 | 1653 | 1776 | 1572 | 1722 | 1659 |
| **Number of amino acids** | 799 | 550 | 591 | 523 | 573 | 552 |
| **PI** | 5.95 | 7.11 | 8.63 | 6.75 | 5.71 | 8.63 |
| **Instability coefficient** | 35.91 | 50.2 | 40.48 | 38.18 | 35.51 | 44.48 |
| **Total average hydrophobicity** | -0.209 | 0.01 | -0.112 | -0.04 | 0.021 | 0.064 |
| **Number of hydrophobic regions** | 156 | 162 | 135 | 142 | 164 | 179 |
| **Number of hydrophilic areas** | 309 | 168 | 194 | 144 | 157 | 162 |
| **Alpha helix (%)** | 21.03 | 24.91 | 27.58 | 34.8 | 29.14 | 30.62 |
| **Extended chain composition (%)** | 24.91 | 19.64 | 28.43 | 14.91 | 18.85 | 18.3 |
| **Irregular curl (%)** | 54.07 | 55.45 | 43.99 | 50.29 | 52.01 | 51.09 |

| **Gene name** | *Fm4CL7* | *Fm4CL8* | *Fm4CL9* | *Fm4CL10* | *Fm4CL11* | *Fm4CL12* |
| --- | --- | --- | --- | --- | --- | --- |
| **Login ID** | KJ531405 | KJ531406 | KJ531407 | KJ531408 | KF994409 | KJ531410 |
| **Length (bp)** | 1701 | 1665 | 1632 | 1758 | 1629 | 1629 |
| **Number of amino acids** | 566 | 554 | 543 | 585 | 542 | 542 |
| **PI** | 8.56 | 8.16 | 8.88 | 7.28 | 5.38 | 5.45 |
| **Instability coefficient** | 36.01 | 30.59 | 35.77 | 40.65 | 36.1 | 37.06 |
| **Total average hydrophobicity** | -0.074 | -0.071 | 0.074 | -0.198 | 0.018 | 0.018 |
| **Number of hydrophobic regions** | 156 | 139 | 162 | 132 | 150 | 150 |
| **Number of hydrophilic areas** | 180 | 162 | 149 | 200 | 151 | 150 |
| **Alpha helix (%)** | 27.92 | 27.44 | 28.18 | 25.47 | 28.23 | 28.23 |
| **Extended chain composition (%)** | 22.61 | 24.73 | 22.1 | 27.18 | 21.59 | 21.59 |
| **Irregular curl (%)** | 49.47 | 47.83 | 49.72 | 47.35 | 50.18 | 50.18 |

Table S2: Primers used for PCR and qRT-PCR assays

| Gene | Forward Primer | Reverse Primer |
| --- | --- | --- |
| *Fm Tu* | AGGACGCTGCCAACAACTTT | TTGAGGGGAAGGGTAAATAGTG |
| *Fm4CL1* | TTCCCGAATTGAATGCTGTTA | TCTGTAAACCTTTGCAGCTC |
| *Fm4CL2* | CTAACAATGGCGGAATCAAG | GAGAAGGTGAAATTCAGAGTAGGA |
| *Fm4CL3* | TTTCCACTTCTCCTTCAACA | TCTGTAAACCTTTGCAGCTC |
| *Fm4CL-like 1* | ATTTTTATCTAGACGGGCGCTCA | GAGGCGAGAAGCGGCACAT |
| *Fm4CL5* | CCAAGAAATCAAAACCCTTTGT | CATGGTCTTTCAGGATACTGTGC |
| *Fm4CL6* | TGCATGTGCTTATTAAATCCTTC | TGATCGTATGGCAATACTACCC |
| *Fm4CL7* | AAACAGAGGGAGATGGAGGAGAGAG | GCAACCGTGTCGCCAAAGC |
| *Fm4CL8* | GGGTACGCTCAAATGCTCAG | CAGCAACAACATCTCCACGA |
| *Fm4CL9* | ACGATGGAGAAATCTGGGTA | ATTGGAGGAGACCGTAAGAA |
| *Fm4CL10* | TGAAAAGAAGCCCATACAGT | TATTTGGAGCCAATACTGAA |
| *Fm4CL11* | TGGAGACTAAAGCAATGCAAGA | ATCTTGTTAAGCCCGGAAGC |
| *Fm4CL12* | CATTCATGGAGACTAAAAGGATG | CACCTTGTTGTATGCCAATCTT |
| *In-Fm4CL-like 1* | TTTTATCTAGACGGGCGCTC | AGCTCCAAATTTTGGGACTTT |
| *NtActin* | CCTGAGGTCCTTTTCCAACCA | GGATTCCGGCAGCTTCCATT |
| *NtHAK1* | ATCCACACCGAGCTTGTTTCAGGA | TGGGTCCAATTCTTCCCACCAAGA |
| *NtAPX* | GCAAGGACATGGAGCAAACAA | CCTCTCTACCAGGGTGAAAGGGAAC |
| *NtCAT* | AATGTGCACTGGCAAACGAG | CCACCCACCGACGAATAAAG |
| *NtABF2* | GATCCGGCGATCAATTCTAA | TTCTCCTCTGCCTCCTTTCA |
| *NtZFP* | ACTGGTCGTTCACACGTCTGTT | AGGCATGTTCAGGTCAAAGTCA |
|  |  |  |
